# Clinical characteristics of nonglaucomatous optic disc cupping

**DOI:** 10.3892/etm.2014.1508

**Published:** 2014-01-28

**Authors:** YI-XIN ZHANG, HOU-BIN HUANG, SHI-HUI WEI

**Affiliations:** 1 Department of Ophthalmology, Hainan Branch of General Hospital of PLA, Sanya, Hainan 572013, P.R. China; 2 Department of Ophthalmology, General Hospital of PLA, Beijing 100853, P.R. China

**Keywords:** optic nerve atrophy, optic disc cupping, nonglaucomatous, retina, human

## Abstract

Pathological optic disc cupping (ODC) is predominantly referred to as glaucoma; however, it is not only glaucoma that leads to pathological optic disc excavation. A number of other nonglaucomatous diseases also result in optic atrophy and excavation of the optic disc. Therefore, in the present study, the etiology of nonglaucomatous optic disc cupping (NGODC) was analyzed and differentiated from glaucomatous optic disc cupping (GODC). The morphology and clinical data of 19 eyes, from 12 patients exhibiting NGODC, were analyzed. Of the 12 cases, none were diagnosed with glaucoma, four presented with optic neuritis, one with Devic’s disease, one with Leber’s hereditary optic neuropathy, two with pituitary adenoma, one with basal ganglia cerebral hemorrhage, one with cilioretinal artery occlusion associated with central retinal vein occlusion, one with central retinal artery occlusion and the remaining patient exhibited optic nerve injuries. The key features that differentiated NGODC from GODC were the color of the optic disc rim and the correlation between visual field defects and the disc appearance. The focally notched disc also aided in distinguishing between the two disorders. The results of the present study indicated that it is critical to acknowledge that nonglaucomatous diseases also lead to ODC and that distinguishing between them is necessary.

## Introduction

Pathological optic disc cupping (ODC) is frequently associated with glaucoma and other less common neuro-ophthalmic conditions (1). Optic atrophy, as a result of numerous nonglaucomatous diseases, may also lead to ODC; however, this has received less consideration. Previous studies have indicated that ~20% of patients were misdiagnosed with glaucoma (2,3). Nonglaucomatous optic disc cupping (NGODC) is predominantly attributed to various optic nerve diseases (4,5); however, may also be a result of retinal diseases (6). Therefore, in the present study, patients with NGODC were analyzed based on data obtained from clinical information and examinations and certain patients were diagnosed with retinal disorders. Distinguishing characteristics between glaucomatous optic disc cupping (GODC) and NGODC are discussed in the present study.

## Patients and methods

### Patients

Medical records of patients who had been diagnosed with NGODC at the Chinese General Hospital of PLA (Beijing, China) between February 2008 and March 2013 were retrospectively reviewed. Informed consent was obtained from all the patients. In total, 19 eyes from 12 subjects (male, 6; female, 6) were analyzed in this study. The patient ages ranged between 8 and 63 (mean age, 39 years; Table I). The medical records of the patients were reviewed and a detailed history of current and past ophthalmic and systemic illnesses was constructed, including visual symptoms on presentation and their duration, medical, family and social histories and any medication previously prescribed.

### Ophthalmic evaluation

Patients underwent a detailed ophthalmic evaluation with particular focus on visual acuity, visual fields, relative afferent pupillary defects, slit-lamp (Topcon Inc., Tokyo, Japan) examinations of the anterior segment, lens and vitreous, intraocular pressure (IOP) (Canon Non-Contact Tonometer; Canon Inc., Tokyo, Japan), ocular movements and fundus evaluation by indirect and direct ophthalmoscopy (also by contact lens if required), as well as additional clinical parameters. Color fundus images (Canon CX-1 fundus camera; Canon Inc., Tokyo, Japan), fluorescein fundus angiography (Heidelberg Retina Tomograph HRT-3; Heidelberg Engineering, Inc., Heidelberg, Germany), optical coherence tomography (Zeiss Cirrus 4000 OCT, Carl Zeiss Meditec, Inc., Dublin, CA, USA), perimetry (Humphrey 740i Field Analyzer, Carl Zeiss Meditec, Inc.) and visual electrophysiology (Roland Electrophysiological diagnostic systems; Roland Consult, Stasche & Finger GmbH, Brandenburg an der Havel, Germany) were also performed.

### Statistical analysis

SPSS 19.0 software (SPSS, Inc., Chicago, IL, USA) was used for statistical analysis. The correlation between IOP and the cup/disc (C/D) ratio was analyzed and compared with analysis of variance. In addition, the data were analyzed using a curve fitting model.

## Results

### Analysis of morphology and clinical data

Of the 12 cases, none were diagnosed with glaucoma, four exhibited optic neuritis, one had Devic’s disease, one had Leber’s hereditary optic neuropathy (LHON), two had a pituitary adenoma, one had a basal ganglia cerebral hemorrhage, one exhibited cilioretinal artery occlusion (CLRAO) associated with central retinal vein occlusion (CRVO), one exhibited central retinal artery occlusion (CRAO) and the remaining patient exhibited optic nerve injuries. The clinical data are described in Table I and selected cases are detailed as follows (the case number refers to the patient number in Table I).

The mean IOP of the affected eyes of the 12 subjects was 14.5±3.8 mmHg (n=19), as compared with the healthy eyes that had a mean IOP of 14.4±1.3 mmHg (n=5). There was no statistically significant difference (F=4.30; P=0.892). The mean C/D ratio in the affected eyes was 0.83±0.10 and 0.38±0.01 in the healthy eyes (F=4.30; P<0.01). There was no correlation between IOP and the C/D ratio (correlation coefficient, 0.184; P>0.05).

### Representative cases

#### Case 2

**Figure 1:**
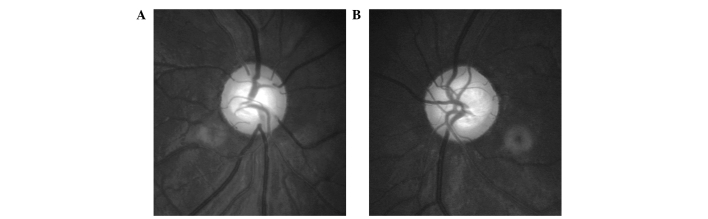
Fundus images of a LHON patient. (A) Right and (B) left retinas exhibit ODC, diffuse rim loss and rim pallor. LHON, Leber’s hereditary optic neuropathy; ODC, optic disc cupping.

An 18-year-old male complained of a sequential decrease in binocular vision over two years. IOPs were 16 mmHg *oculus dexter* (OD) and 14 mmHg *oculus sinister* (OS). The patient was diagnosed with LHON that had been caused by a 11778A mtDNA mutation. Bilateral optic disc appearance was characterized via diffuse excavation of the optic cup and rim pallor (Fig. 1). A central scotoma was the predominant visual field defect observed.

#### Case 3

**Figure 2:**
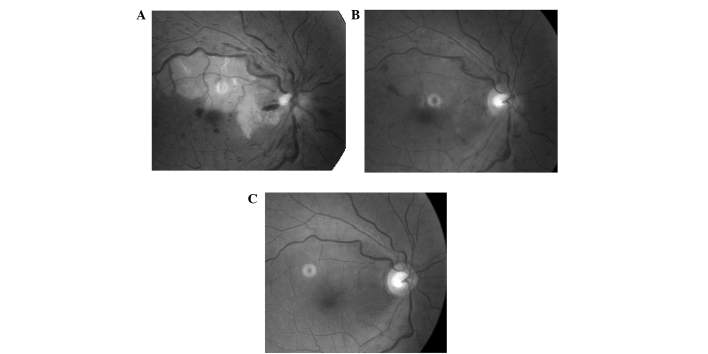
Fundus images of a patient exhibiting CLRAO and CRVO. (A) Retinal manifestation observed at the initial visit, exhibiting retinal vein dilation, retinal hemorrhage and retinal infarct corresponding to the region of CLRAO. (B) Fundus image one month after the initial visit. (C) Fundus manifestation ten months after the initial visit. Gradual diffuse excavation of the optic disc between A and C is apparent. CLRAO, cilioretinal artery occlusion; CRVO, central retinal vein occlusion.

A 62-year-old female with CLRAO and CRVO of the right eye exhibited increasing excavation of the cup with superior and inferior rim loss. Temporal rim pallor was identified in the follow-up examinations (Fig. 2). IOP was 15 mmHg OD.

#### Case 5

**Figure 3:**
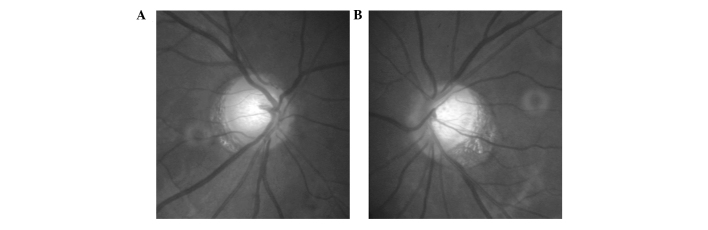
Fundus images of a patient exhibiting a hemorrhage in the left basal ganglia obtained 13 months after the onset of bleeding. (A) Nasal rim loss was apparent in the right eye, predominantly in the superior nasal region. (B) Rim loss in the left eye was located in the temporal region.

A 31-year-old female complained of a visual field defect of the right eye over a six-month period. IOPs were 14 mmHg OD and 14 mmHg OS. A magnetic resonance imaging scan revealed a hemorrhage in the left basal ganglia, which also involved the left cerebral peduncle and basal ganglia. Left temporal and right nasal disc pallor and rim loss were observed with the corresponding visual field defect of right homonymous hemianopia (Fig. 3).

#### Case 7

**Figure 4:**
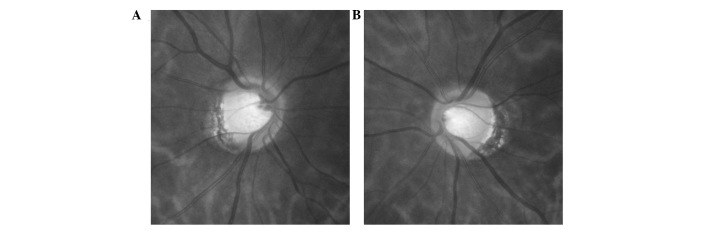
Fundus images of a patient exhibiting optic neuritis. (A) Right and (B) left retinas show ODC, however, optic disc pallor was apparent in the left eye. The patient was diagnosed with physiological ODC and left optic atrophy. ODC, optic disc cupping.

A 47-year-old male experienced decreased vision in both eyes for ten months. The patient had been diabetic for three years. IOPs were 17 mmHg OD and 16 mmHg OS. Optic disc excavation and disc pallor were identified in both eyes, but was more serious in the left eye (Fig. 4). The patient exhibited congenital physiological ODC with disc pallor as a result of optic atrophy following optic neuritis.

## Discussion

A strong association was observed between neuro-ophthalmic diseases and pathological ODC in the present study. In addition to glaucoma, other retinal diseases contribute to NGODC (6). Retinal and optic nerve head diseases are often associated with NGODC, including arteritic anterior ischemic optic neuropathy (AION; 7–10), which is the most common cause despite being rarely observed in China. In the present study, one patient with CLRAO and CRVO also exhibited NGODC, which to the best of our knowledge, is the first such case to be reported in literature. Furthermore, optic neuropathies may lead to NGODC, including LHON, autosomal dominant hereditary optic atrophy, optic neuritis, toxic optic neuropathy (e.g. methanol poisoning) and traumatic neuropathy. In addition, chiasmatic diseases and other diseases, including anterior segmental optic nerve compression, cerebrovascular diseases, radiation optic neuropathy and carotid artery stenosis, occasionally lead to NGODC (6). Previous studies have focused on other causes, such as retrograde transsynaptic degeneration of retinogeniculate axons following periventricular leukomalacia (11), physiological cupping and nonglaucomatous optic atrophy, which are additional manifestations of NGODC.

A normal optic disc usually forms a vertical oval shape with a vertical diameter that is 7–10% greater than the horizontal diameter. The optic cup is horizontally oval, thus, the horizontal C/D ratio is larger than the vertical C/D ratio. In healthy individuals, the median C/D ratio value is <0.3 and the difference in the C/D ratios between the fellow eye is <0.2. Therefore, a ratio greater than 0.6 is indicative of ODC. The color of a normal rim is orange due to the presence of capillaries. In the majority of cases, a normal width follows the inferior, superior, nasal, temporal (ISNT) rule, thus, the neuroretinal rim is broadest in the inferior disc region, followed by the superior disc region, the nasal disc area and finally the temporal disc portion. However, violation of the ISNT rule also occurs in large optic disc cups of nonglaucomatous origin, with a greater frequency in the pediatric population (12).

Changes in the optic disc as a result of glaucoma include focal or concentric enlargement, where the vertical diameter change is disproportionate to the horizontal change. Additional traits include deepened excavation, increased exposure of the lamina cribrosa, diffuse rim loss, wedge-shaped nerve fiber layer defects, flame-shaped disc hemorrhages and beta zones of parapapillary atrophy in accordance with nerve fiber layer defects (13).

Several key differences were observed between NGODC and GODC (14–16). Firstly, the color of the rim is the most important. The rim of NGODC often exhibits pallor, while the rim in GODC is pink. However, differentiating between NGODC and GODC according to rim color is very difficult in end-stage glaucoma when the C/D ratio is ~1.0. Secondly, the presence of focal or diffuse rim loss is important. Focal rim loss is predominantly associated with glaucoma, while eyes with nonglaucomatous diseases are often characterized by diffuse rim loss. Although focal rim loss may occasionally be present in NGODC, total loss of the disc rim is never observed. Thirdly, there is a high correlation between visual field defects and disc changes in GODC, but a marginal correlation between these parameters in NGODC (4,14,15). Fourthly, IOP may or may not be within the normal range. Fifthly, ODC is apparent prior to visual field defects in GODC. Visual acuity decreases markedly in NGODC with apparent visual field losses, but with marginal associated changes in the optic cup. Finally, peripapillary atrophy is an increasingly common observation in GODC as compared with NGODC.

Physiological cupping is a congenital disorder of optic cupping, which is caused by the scleral optic canal and pronounced glial atrophy of Bergmeister’s papilla. GODC is a type of ascending optic nerve atrophy that is associated with the loss of retinal ganglion cell axons. These changes extend anterogradely (upwards) along the pathological axons, which is followed by neuroretinal rim loss and increased exposure of the lamina cribrosa. In eyes exhibiting glaucomatous damage, rim loss is predominantly identified in the inferior and superior disc regions, thus, cupping is prone to vertical expansion. Focal or diffuse rim loss is associated with the distribution of nerve fibers and the impaired area. NGODC, resulting from optic nerve diseases, is an abnormality of retrograde (descending) optic atrophy, the mechanisms of which include prelaminar tissue atrophy, postlaminar tissue fibrosis, the shrinkage of fiber longitudinales, glial hyperplasia, bowing back of the lamina cribrosa following loss of support and damage to the laminar neurological and connective tissue caused by ischemia (7).

Eyes with non-arteritic AION (NA-AION) do not develop ODC. Patients with NA-AION usually exhibit a small or no optic cup, which may contribute to the pathology (7). However, the damage and atrophy of retinal ganglion cell axons in NA-AION is not as serious as that observed in arteritic AION, in which occlusion of the posterior ciliary arteries is a key event in pathogenesis and results in greater diffuse damage (7).

In conclusion, the key differentiating features between NGODC and GODC are the optic disc rim color and the correlation between visual field defects and the disc appearance. Focal rim loss also aids with distinguishing between the two diseases. However, methods of distinguishing NGODC from GODC should rely on patient history, visual field assessment and clinical data. Physicians and ophthalmologists are required to be vigilant to uncommon and potentially threatening forms of NGODC.

## Figures and Tables

**Table I:** Clinical data of 12 patients with NGODC.

Case no.	Gender	Age (years)	Diagnosis	Eye	Duration of disease^a^	Morphology of cupping	BCVA^b^	Visual field defect
1	F	41	Neuromyelitis optica	OS	2 years	Diffuse	OD 0.2, OS FC/20 cm	Central scotoma
2	M	18	LHON	OU	2 years	Diffuse	OD 0.1, OS 0.05	Central scotoma
3	F	62	CLRAO, CRVO	OD	10 months	Diffuse	OD 0.15, OS 0.25	Inferior defect
4	M	26	Pituitary adenoma	OU	10 months	Bilateral nasal (more serious OD)	OD 0.8, OS 0.8	Bitemporal hemianopsia
5	F	31	Cerebral hemorrhage	OU	13 months	Temporal OS, nasal OD	OD 1.0, OS 1.0	Bilateral homonymous hemianopia in the right side
6	M	55	Optic neuritis	OD	1 year	Diffuse	OD 0.3	Diffuse defect
7	M	47	Optic neuritis	OU	10 months	Diffuse	OD 1.0, OS 0.12	Diffuse defect OS, more serious superiorly. Superior defect OD
8	F	8	Optic neuritis	OU	6 months	Diffuse	OD 0.8, OS 0.8	Diffuse defect, more serious peripherally
9	F	48	Optic neuritis	OS	1 year	Diffuse	0.1	Unknown
10	M	39	Pituitary adenoma	OU	2 years	Diffuse	OD 1.2, OS HM before eye	Diffuse defect OS Nasal hemianopia OD
11	F	30	Optic nerve injury	OU	2 years	Diffuse	NLP, OU	Unavailable
12	M	63	CRAO	OD	3 months	Diffuse	0.1	Diffuse defect

a Indicates the time period between the clinical discovery of ODC and onset of the disease.

b Indicates BCVA post follow-up.

NGODC, nonglaucomatous optic disc cupping; F, female; M, male; LHON, Leber’s hereditary optic neuropathy; CLRAO, cilioretinal artery occlusion; CRVO, central retinal vein occlusion; CRAO, central retinal artery occlusion; OS, *oculus sinister*; OU, *oculus uterque;* OD, *oculus dexter*; FC, finger counting; HM, hand movement; NLP, no light perception; BCVA, best corrected visual acuity.

## Abbreviations

ODC: optic disc cupping
GODC: glaucomatous optic disc cupping
NGODC: nonglaucomatous optic disc cupping
IOP: intraocular pressure
C/D: cup/disc ratio
LHON: Leber’s hereditary optic neuropathy
CLRAO: cilioretinal artery occlusion
CRVO: central retinal vein occlusion
CRAO: central retinal artery occlusion
OD: *oculo dextro*
OS: *oculus sinister*
AION: anterior ischemic optic neuropathy
ISNT: inferior, superior, nasal, temporal
NA-AION: non-arteritic anterior ischemic optic neuropathy
